# Recent changes in sociodemographic characteristics, dietary behaviors and clinical parameters of adults receiving food assistance in France

**DOI:** 10.1186/s12889-016-3443-9

**Published:** 2016-08-12

**Authors:** Katia Castetbon, Dorothée Grange, Gaëlle Guibert, Michel Vernay, Hélène Escalon, Catherine Vincelet

**Affiliations:** 1 Unité de Surveillance et d’Epidémiologie Nutritionnelle (USEN), Institut de Veille Sanitaire (InVS), Université Paris 13, Centre de Recherche en Epidémiologie et Statistiques, Bobigny, France; 2 Université Libre de Bruxelles, Ecole de Santé Publique, Centre de Recherche en Epidémiologie, Biostatistiques et Recherche Clinique, Route de Lennik 808, 1070 Bruxelles, Belgium; 3 Observatoire de Santé Ile-de-France, Paris, France; 4 Unité Cardiorespiratoire Diabète (UCARED), Institut de Veille Sanitaire (InVS), Saint-Maurice, France; 5 Institut National de Prévention et d’Education pour la Santé, Saint-Denis, France

**Keywords:** Dietary behavior, Food assistance, France, Obesity, Repeated survey

## Abstract

**Background:**

In 2004–2005, a survey carried out on food recipients in France revealed an alarming nutritional situation. In 2011–2012, and using a protocol similar to that of 2004–2005, our objective was to update the description of sociodemographic characteristics, dietary behaviors and clinical parameters of food assistance recipients and to analyze changes since 2004–2005.

**Methods:**

Both surveys included multistage random sampling of adults benefitting from structures that supply food pantries and charitable grocery stores. Data on sociodemographic characteristics and dietary behaviors were collected along with weight, height and blood pressure measurements. Comparisons between the 2004–2005 (*n* = 883) and 2011–2012 (*n* = 1,058) survey observations were made, adjusting for socio-demographic changes which had occurred in the meantime.

**Results:**

Since 2004–2005, proportions of food recipients ≥55 years (13.1–19.1 %), born in France (29.2–36.8 %) and employed (5.5–11.7 %) have increased; food insufficiency has decreased (95–74 %). For over half of the recipients, canned (52.4 %) and non-perishable (50.9 %) foods were obtained only from food assistance. Frequency of consumption significantly increased even after adjustment for socio-demographic changes; this was the case for dairy products (for twice a day consumption, 30.2–36.4 %), fruits and vegetables (three times a day, 7.8–13.9 %), and meat, eggs and fish (twice a day, 9.4–19.2 %). In 2011–2012, 15.6 % of men and 36.0 % of women were obese, while 44.5 and 35.1 % had high blood pressure, respectively.

**Conclusions:**

Between 2004–2005 and 2011–2012 in France, consumption of staple foods has been slightly improved in food assistance recipients. However, prevalence of cardiovascular risk factors remains high, which underlines the need for long-term efforts at better quality of foods delivered.

## Background

Access to nutritionally adequate, safe foods, as well as the ability to acquire such foods in a socially acceptable manner, form the basis for food security [1]. In high-income countries, this may be compromised in persons coping with economic difficulties. Indeed, the proportion of population suffering from food insecurity has been estimated at between 5 % in 2012 in Korea [2] and 15 % in 2004–2005 in New Zealand [3] and in 2013 in the United States [4]. Means for reducing food insecurity include financial, housing and food assistance. For instance, benefiting from the U.S. Supplemental Nutrition Assistance Program (SNAP) has been estimated to reduce food insecurity: after one year continuing on SNAP, the odd of very low food security was 28 % lower than among those that left the program before 30 days [5]. After 2 years, the difference was 45 %. However, such observations are not necessarily generalized to all food assistance systems due to various quantity, quality and accessibility of food provided, and to variable characteristics of those who seek food assistance.

Some western countries have set up systems to deliver food assistance via state and/or non-governmental organizations which may later be financially supported by the state, as in the European Union, via the Most Deprived Persons Program (MDP) [6]. Food assistance is delivered in the form of free meals for immediate consumption (especially for the homeless), free food parcels and “social groceries” that require a small financial participation by the recipients [7]. Regular evaluation of recipient characteristics, their dietary behavior and nutritional status, is highly useful for adapting foods to be delivered and organizing food assistance. However, apart from estimating the prevalence of food insecurity among food aid recipients [8, 9], comprehensive evaluations are rare, especially in Europe [10].

In France, as in Canada [7], food assistance may take on different forms, and is present mainly in urban centers. In 2004–2005, we carried out a first assessment of sociodemographic and nutritional characteristics in food aid recipients in four French urban zones (Paris, Marseille, Dijon and Seine-St-Denis) [11]. Named “Abena” (*Alimentation et état nutritionnel des bénéficiaires de l’aide alimentaire*), this survey revealed an alarming nutritional situation that we reported to government authorities. Measures were then taken to provide better food quality and greater quantities (especially of fruits, vegetables and fish) and to improve the delivery distribution system (transport and storage). Furthermore, the characteristics of individuals requiring food assistance may have changed since the first Abena study. Often used in surveillance systems, repeated cross-sectional surveys help assess and understand such changes in the same source population but without the cohort limitations such as selection bias [12]. In 2011–2012, using a protocol similar to that of 2004–2005, our aims were to update the description of sociodemographic characteristics, dietary behaviors and clinical parameters of food assistance recipients, and to analyze changes between the two periods.

## Methods

### Sampling

In 2011–2012, the survey was carried out in three cities, Paris, Marseille and Dijon, and three departments surrounding Paris (Seine-St-Denis, Val-de-Marne and Hauts-de-Seine). They were purposely chosen for their population characteristics such as age distribution, unemployment proportions, or migration characteristics, based on Census information. In total, 226 food banks that deliver food in the form of parcels and social groceries were listed several weeks before data collection, along with the number of recipients for each bank in 2010. Such a list led to highly variable situations regarding eligibility criteria of people seeking food assistance, frequency of distribution, type of foods delivered etc. We used a two-stage sampling scheme. The first stage was a random selection of 62 food banks, proportionally allocated based on the number of recipients; four structures refused to participate. The second stage of selection was based on a random number list that defined the first person to be selected in the survey during the days the interview was carried out. Inclusion criteria were: age 18 or over; no other household member already included; capacity to understand reasons for the survey and to answer the questionnaire in French, alone or with help from an interpreter; recipient of food assistance (i.e., the person who was registered and came to the food distribution, even if the food was delivered for his/her family).

In 2004–2005, the survey was carried out in Paris, Marseille, Dijon and Seine-St-Denis using the same sampling scheme and inclusion criteria. Details have already been published [11].

### Data collection at food assistance centers

In both surveys, data collection was carried out between November and April, the period during which food assistance is highest in France. Trained dieticians collected information on standardized questionnaires that included socio-demographic and economic characteristics, dietary behavior and food supply, use of food assistance, food insecurity and health characteristics. If possible, interviews were carried out in separate rooms for confidentiality and accuracy.

In 2004–2005 and 2011–2012, socio-demographic and economic data included age, gender, marital status, number of children, place of birth, housing type, education, employment and household income, including social assistance. Dietary behavior included frequency of usual food consumption on a daily basis (“bread, toast, breakfast cereal”; “rice, pasta, potatoes, semolina”; “vegetables (except for potatoes)”; “fruits, including 100 % fruit juice”; “dairy”; “meat, poultry, eggs”) with eight categories, from “never” to “4 times a day or more”. Consumption frequency of “seafood, including canned seafood” and “legumes” was proposed on a weekly basis, with the 7 categories from “never” to “4 times a week or more”.

The sources of food supplies were as follows: “market”, “small supermarket”, “super/hypermarket”, “low-cost store”, “food assistance”, “donations (out of food assistance)” and “market or garbage recovery” for a given list of foods. We identified, among those obtaining such foods, subjects who declared food assistance as their only food source. In addition, history and past-year frequency of food assistance were collected, as well as perception of food assistance quality and organization. In 2011–2012, the USDA 18-item Food Security Survey Module (FSSM) was used to assess food insecurity [13]. In both 2004–2005 and 2011–2012, food insufficiency was assessed using one question: “Which of these statements best describes the food eaten in your household in the last 12 months: − Enough of the kinds of food we want to eat; − Enough but not always the kinds of food we want; − Sometimes not enough to eat; − Often not enough to eat; − Do not know or Refused.” [14].

### Body weight status and blood pressure measurements

At the end of the interview at the food assistance center, participants were invited to undergo biochemical and clinical examinations at a municipal or health insurance (CnamTS) health center. Recipients who agreed to this signed an informed consent and, a few days later, underwent a health examination that included measurements of anthropometry and blood pressure, information on drug intake and fasting blood sampling. Measurement of blood pressure and anthropometry at the food assistance center was also proposed whether or not they finally underwent the examination at a health center. The same procedures and devices were used at health centers and food assistance structures, similarly to 2004–2005 [11].

For blood pressure measurements, Omron® M5-I was used according to a standardized protocol: after a 5-min rest, the first measurement was performed on the right arm and then the left. A third measurement was made on the arm with the highest values. The highest values (systolic and diastolic blood pressure) on this arm were retained for statistical analyses. Weight and height measurements were also standardized using identical devices at all centers, to the nearest 0.1 kg with Seca® Bellissima 841 scales and to the nearest 0.5 cm with Soehnle® ultrasound gauges, respectively.

### Statistical analyses

Weekly and daily frequencies of food consumption were computed for “starchy foods” as the sum of “bread, toast, breakfast cereal”, “rice, pasta, potatoes, semolina” and “legumes”; for “fruits and vegetables” as the sum of “vegetables except for potatoes” and “fruits, including 100 % fruit juices”; and for “meat, fish, eggs” as the sum of “meat, poultry, eggs” and “seafood”. Descriptions were drawn up by grouping together certain consumption frequencies according to the distribution observed for a given food group (see Figures for details). Food insecurity prevalence was computed according to recommendations for use of the 18-item FSSM [15]. For instance, in households with one or more children, “severe food insecurity” was defined by a score of 8–18; “moderate food insecurity” by a score of 3–7, and “no food insecurity”, by a score of 0–2. In households without child, the score ranges were: 6–10, 3–5 and 0–2, respectively. Individual scores were also used for food insecurity in children [15].

For anthropometry and blood pressure, statistical analyses were carried out using measurements obtained at a health examination center and, for subjects who declined to undergo the complete health examination, measurements at a food assistance center. World Health Organization (WHO) cut-offs were used to define high blood pressure (140 mmHg and 90 mmHg for systolic blood pressure and diastolic blood pressure, respectively [16]) irrespective of drug intake, and to define body weight status according to body mass index (BMI, weight / height^2^) [17]: thinness: BMI <18.5; normal weight: BMI ≥18.5 and <25.0; overweight: BMI ≥25.0 and <30.0; and obesity: BMI ≥30.0.

In all analyses performed using Stata® V.12, the complex sampling scheme (food assistance centers, and then individuals) was taken into account, along with unequal probabilities of inclusion using “svyset” and “svy”. Analyses were carried out for the entire 2011–2012 sample, and for the same sample, but limited to the same four zones as in the 2004–2005 survey so as to assess changes over time in the source population. Rao-Scott chi-squared tests were used to compare socio-demographic and nutrition characteristics between 2004–2005 and 2011–2012 surveys in the same four geographic zones.

In order to assess the potential effect of socio-demographic changes upon changes observed between the two surveys for food assistance as the main source of food, consumption frequencies, BMI and BP, multivariate logistic regressions were also performed using multinomial models if the outcome included more than two categories. In addition to the survey year variable used to assess the statistical significance of changes between the two surveys, covariates included in the models were: sex, age group, family status, birthplace, education and employment status. They were used as they are described in the first part of the results (Table 1). For that purpose, food consumption frequencies were merged into a lower number of categories as follows: starchy foods and fruit and vegetables as “<3 times a day”, “3 times” and “>3 times a day”; dairy as “< twice a day”, “twice a day” and “> twice a day”; meat, fish and eggs as “<once a day”, “once a day” and “>once a day”. The base outcome was set to the lowest frequency category. A *P*-value < 0.05 was considered statistically significant.

**Table 1:** Socio-demographic and living conditions (weighted %) of adults receiving food assistance in France in 2004–2005 and 2011–2012

		Paris, Marseille, Dijon, Seine-St-Denis			Same + Val-de-Marne, Hauts-de-Seine
		2004–2005 *n* = 883	2011–2012 *n* = 1,058	*P*	2011–2012 *n* = 1,575
Gender	n	883	1,058	NS	1,575
Male		20.6	19.9		24.4
Female		79.4	80.1		75.6
Age	n	877	1,057	<0.05	1,574
< 25 y		4.5	5.3		5.7
25–34 y		24.1	22.8		21.9
35–54 y		59.3	52.8		51.9
55–64 y		9.8	11.7		13.7
≥ 65 y		2.3	7.4		6.8
Place of birth	n	882	1,047	<0.01	1,564
France		29.2	36.8		38.7
Eastern Europe		3.6	3.1		2.5
Sub-Saharan Africa		13.3	18.7		19.3
North Africa		48.5	34.1		32.2
Other		5.3	7.4		7.3
Marital status	n	880	1,057	<0.05	1,574
Married or living with a partner		50.6	38.7		36.2
Single		49.4	61.3		63.8
Number of children	n	882	1,058	<0.05	1,575
0		20.6	33.4		36.1
≥ 1		79.4	66.6		63.9
Type of dwelling	n	882	1,049	NS	1,566
House/flat; housed by family		87.9	90.5		88.0
Shelter		8.8	6.3		8.0
Homeless		3.4	3.2		4.0
Current job status	n	874	1,042	<0.001	1,558
Working		5.5	11.7		10.8
Not working		74.5	80.0		80.5
Illegal immigrant		20.0	8.3		8.7
Education diploma	n	876	1,036	<0.05	1,551
None or primary		59.7	48.2		46.5
High school		23.3	31.6		32.8
High school diploma		10.8	12.5		12.8
University		6.2	7.7		7.9
Food insufficiency	n	873	999	<0.001	1,503
Sometimes/often not enough to eat		45.8	29.7		31.5
Enough to eat but not always the kind of foods wanted		50.3	44.4		43.4
Enough to eat		3.9	25.8		25.1
History of food assistance use	n	883	1,058	<0.05	1,575
0–6 months		41.4	28.4		29.5
7–12 months		6.1	7.2		6.7
13–24 months		17.1	16.4		15.7
> 24 months		35.4	48.0		48.1

*NS* non-significant

## Results

In 2011–2012, at food assistance centers delivering parcels and social groceries, 3,777 individuals were invited to participate in the survey and 1,575 answered the questionnaire (participation rate: 41.7 %). Lack of time was the most frequent reason for refusal (82.9 %). The number of participants was equally distributed across geographical zones: 250 in Marseille, 270 in Dijon, 297 in Paris, 266 in Hauts-de-Seine, 241 in Seine-St-Denis and 251 in the Val-de-Marne.

In 2011–2012, more than three-fourths of food assistance recipients were female, similar to 2004–2005 (Table 1). The numbers of subjects living in a house or flat, or housed by family members, were also comparable in the two surveys. Most food recipients were 35–54 years old, but the proportions of 55–64 year olds and of those over 65 were higher in 2011–2012 than in 2004–2005 (Table 1). In 2011–2012, more than one-third were born in France. Since 2004–2005, this proportion has increased, whereas the proportion of those born in North Africa has decreased. Two-thirds of food recipients had at least one child, but six out of ten were single: 34 % were one-parent families. In 2011–2012, around 12 % of food recipients were working, which was twice that reported in 2004–2005. In addition, half of them went beyond primary school, a proportion that has increased since 2004-2005 (Table 1).

In 2011–2012, food insufficiency was present in three-fourths of individuals, either in quantity (around 30 %) or quality (44 %). Such proportions have significantly decreased since 2004–2005: at that time, 95 % reported food insufficiency (Table 1). In 2011–2012, in the entire sample, the prevalence of household food insecurity using the 18-item FSSM was estimated for 1,287 subjects and the prevalence of child food insecurity for 758 subjects (estimated only among those living in households with children). A total of 43.5 % of households in which food recipients resided experienced severe food insecurity, and 31.1 %, moderate food insecurity. Moreover, 9.1 % of children of food recipients experienced severe food insecurity and 33.9 %, moderate food insecurity.

In 2011–2012, proportions of socio-demographic characteristics and food insecurity in the entire sample were similar to those in the sample restricted to the same zones as in the 2004–2005 survey (Table 1).

### Use of food assistance

**Fig. 1:**
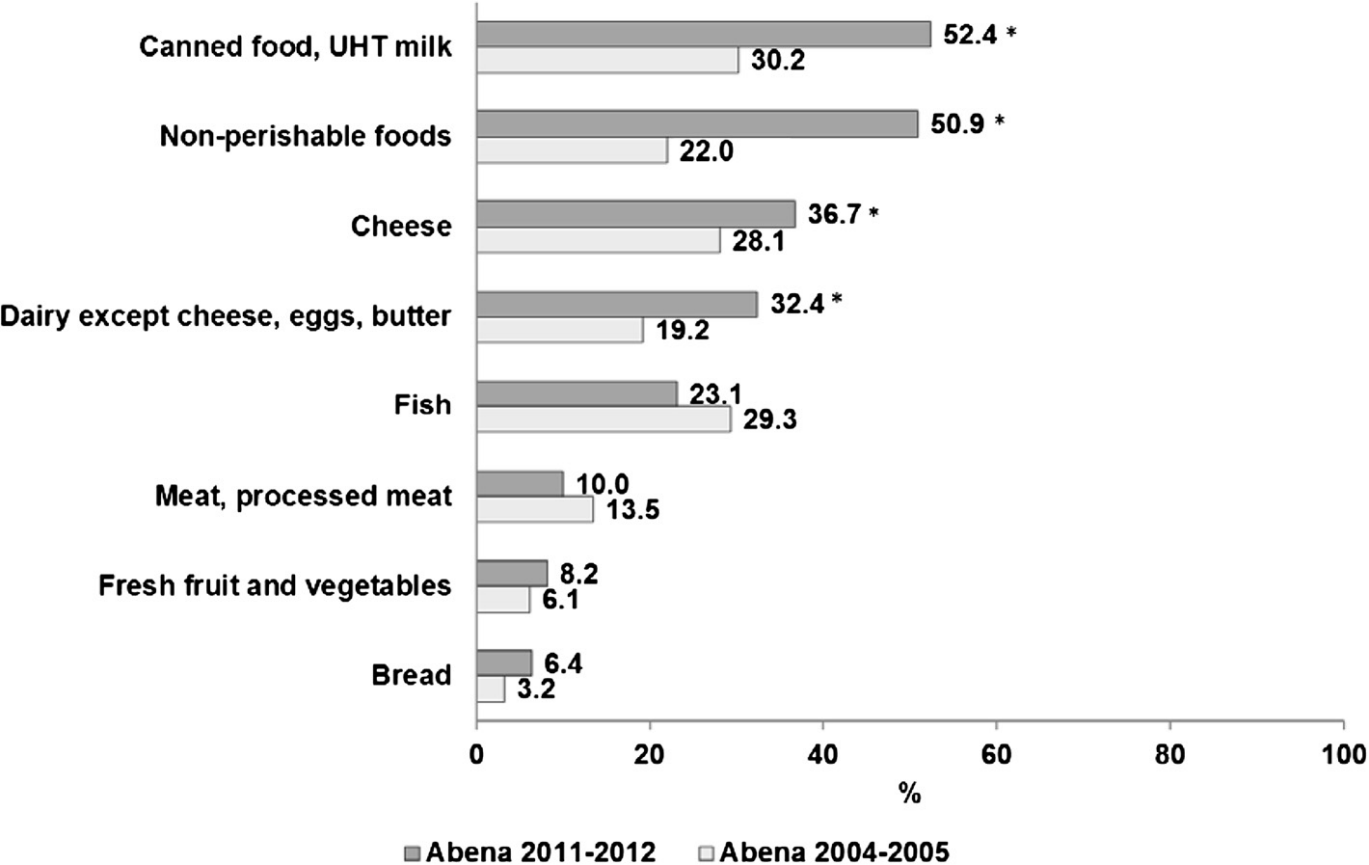
Food assistance as the exclusive source of food supply (weighted %) in France in 2004–2005 and 2011–2012

In 2011–2012, about half of the food recipients had been receiving food assistance for at least 2 years (49.3 %), while 28.7 % had been benefitting from it for less than 6 months. In 2004–2005, figures were 35.4 % and 41.4 %, respectively (*P* < 0.05) (Table 1). For half of the recipients, food assistance was the only source of canned foods, UHT milk and non-perishable foods (pasta, rice, cereals, sugar, condiments, etc.) (Fig. 1). These proportions have dramatically increased since 2004–2005 (*P* < 0.0001) (Fig. 1). For cheese (*P* = 0.02) and other dairy products (*P* = 0.0004) (Fig. 1), food assistance was the only source, again in higher proportions than in 2004–2005. In contrast, use of food assistance as the exclusive source of meat, processed meats, fresh fruit and vegetables and bread remained at less than 15 %, with no significant change since 2004–2005. Fish was obtained through food assistance exclusively in 23 % of food recipients, similarly to 2004–2005 (Fig. 1). When adjusting for socio-demographic characteristics, all results were similar, except for meat and processed meats, for which the survey year became statistically significant (*P* = 0.005).

### Food consumption frequency

**Fig. 2:**
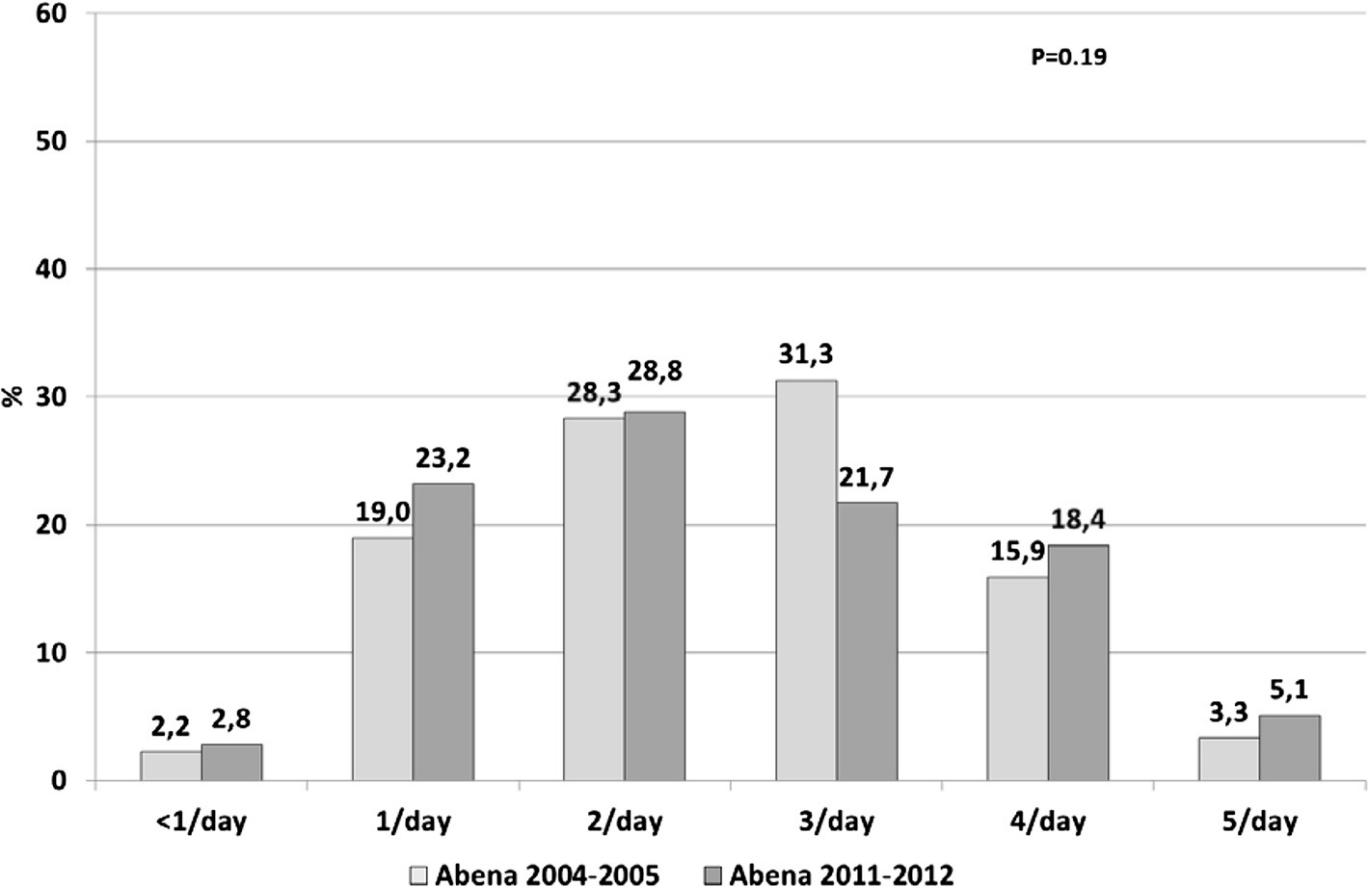
Frequency of starchy food consumption (weighted %) in persons receiving food assistance in France in 2004–2005 and 2011–2012

**Fig. 3:**
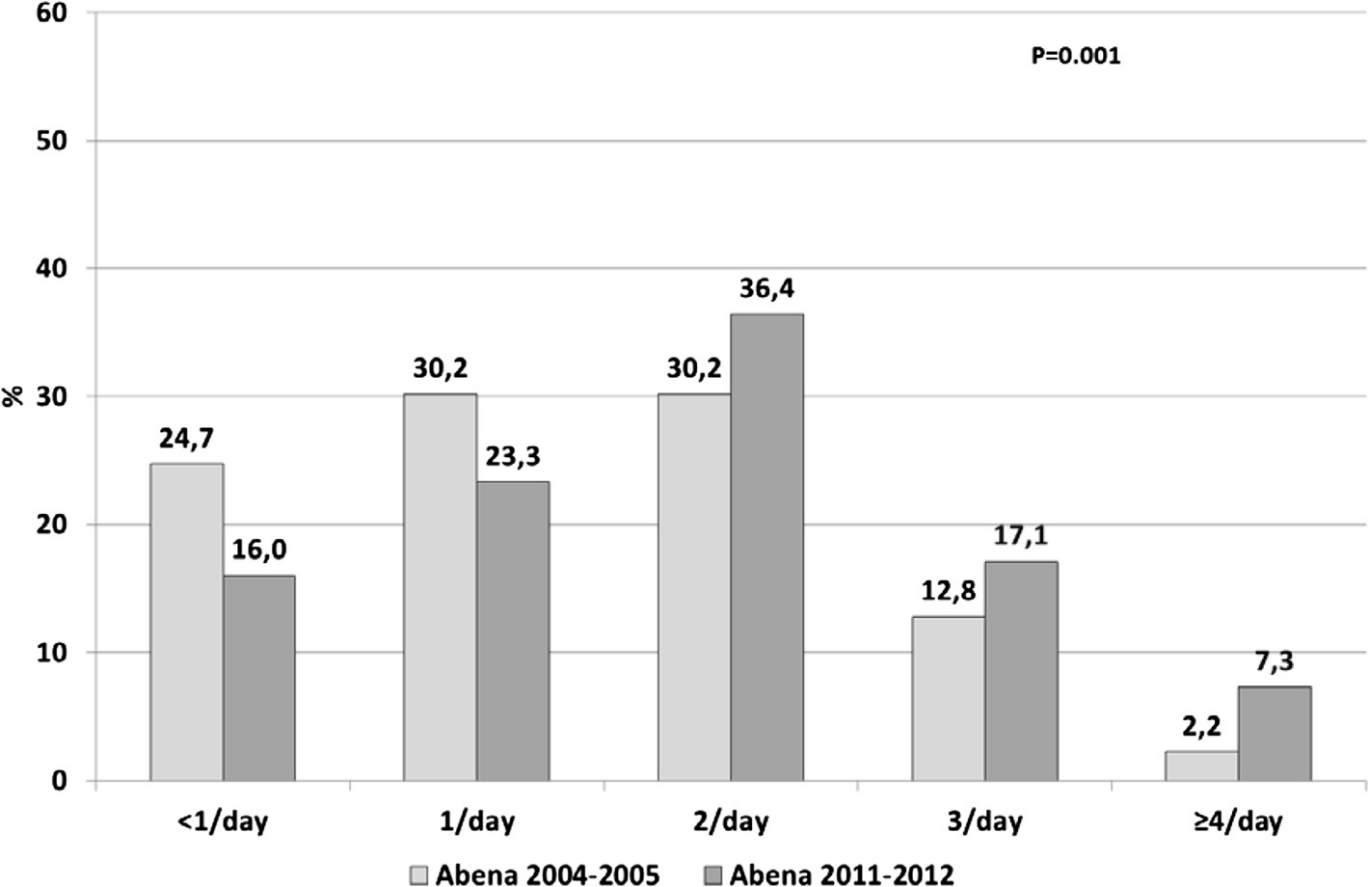
Frequency of dairy consumption (weighted %) in persons receiving food assistance in France in 2004–2005 and 2011–2012

**Fig. 4:**
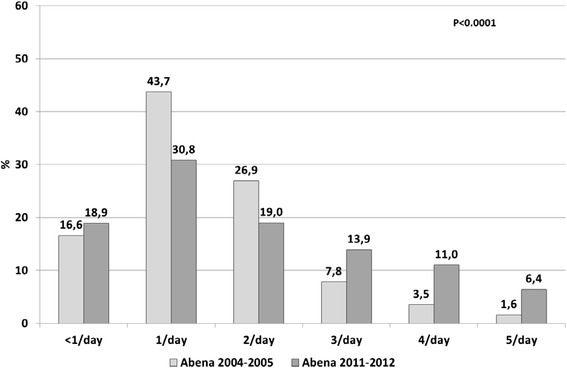
Frequency of fruit and vegetable consumption (weighted %) in persons receiving food assistance in France in 2004–2005 and 2011–2012

**Fig. 5:**
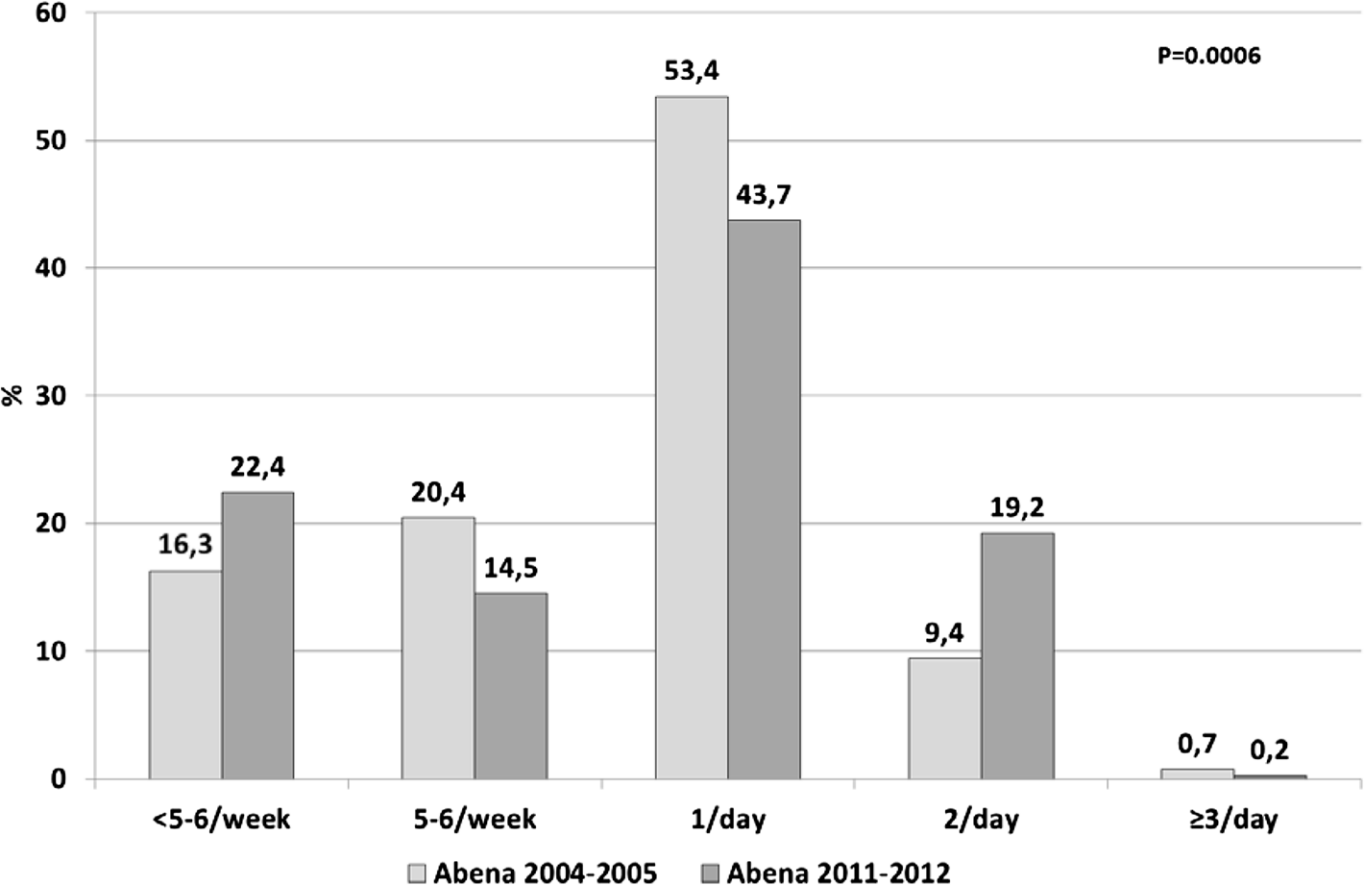
Frequency of meat, fish and egg consumption (weighted %) in persons receiving food assistance in France in 2004–2005 and 2011–2012

**Fig. 6:**
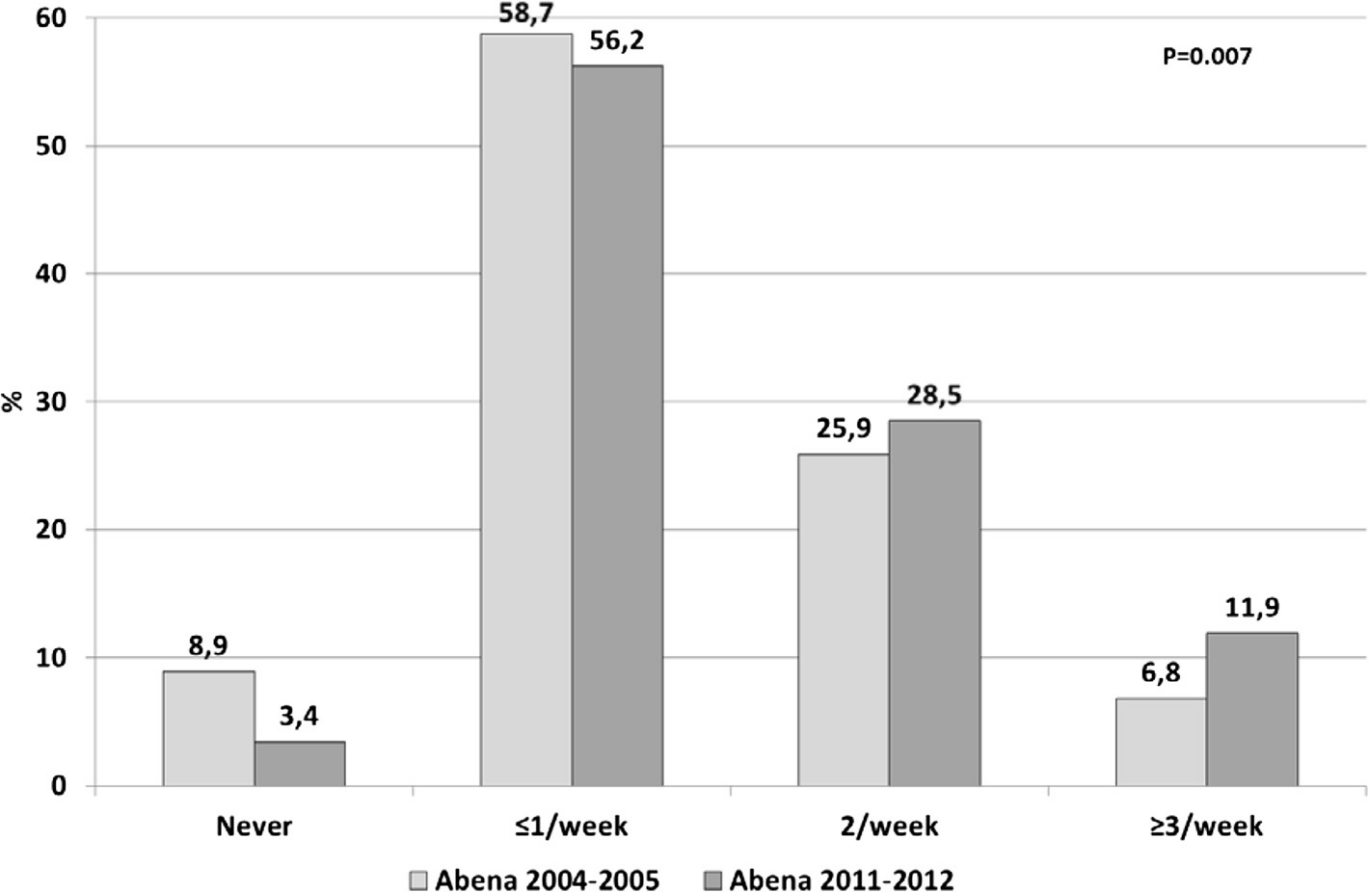
Frequency of fish consumption (weighted %) in persons receiving food assistance in France in 2004–2005 and 2011–2012

In 2011–2012, starchy foods were consumed daily by 97.2 % of food recipients (Fig. 2), dairy products by 84.1 % (Fig. 3), fruit and vegetables by 81.1 % (Fig. 4) and meat, fish and eggs by 63.1 % (Fig. 5). Frequency of starchy food consumption did not change since 2004–2005 (*P* = 0.19) (Fig. 2). In contrast, dairy product consumption increased, toward frequencies of twice a day, three times a day and ≥4 times a day (*P* = 0.001) (Fig. 3). Fruit and vegetable consumption also significantly increased (*P* < 0.0001), especially for frequencies of 3 times a day or more, while once-a-day and twice-a-day frequencies decreased (Fig. 4). Food recipients who ate meat, fish and eggs 5–6 times a week or once a day were proportionally fewer in 2011–2012 than in 2004–2005, while one out of five consumed them twice a day in 2011–2012, compared to one out of ten in 2004–2005 (*P* = 0.0006) (Fig. 5). In 2011–2012, fish was consumed once a week or less by half of the food recipients (Fig. 6). Frequencies of twice a week and three times a week or more increased since 2004–2005 (*P* = 0.007). Changes over survey years were still statistically significant when adjusting for socio-demographic characteristics (data not shown). In addition, food consumption frequencies estimated in 2011–2012 were similar to the 2004–2005 survey in the entire sample and in the sample limited to the common geographical zones.

### Body weight status and blood pressure

Among subjects who answered the questionnaire, 908 (57.6 %) were measured for weight and height and 1,000 (63.5 %) for blood pressure in 2011–2012; in 2004–2005, numbers were 539 (61.0 %) and 579 (65.6 %), respectively. In 2011–2012, three-fourths of the women and more than half of the men were overweight or obese (Table 2). Among them, 37 % of females and 15 % of males were obese. Moreover, one-third of the women and half of the men were found to have high blood pressure (in addition, 5.3 % declared receiving drugs against hypertension). Proportions of obesity and of high blood pressure have increased since 2004–2005, although not significantly, except for blood pressure in women (Table 2). When adjusting for socio-demographic characteristics, the increase in obesity between 2004–2005 and 2011–2012 was also statistically significant in women (*P* = 0.02), but was no longer significant for hypertension in women.

**Table 2:** Measured body weight status and elevated blood pressure (weighted %) in persons receiving food assistance in France in 2004–2005 and 2011–2012

		Paris, Marseille, Dijon, Seine-St-Denis						Same + Val-de-Marne, Hauts-de-Seine	
		2004–2005		2011–2012		*P*		2011–2012	
		Men	Women	Men	Women	M	W	Men	Women
Body mass index (%)	n	145	394	177	491	NS	NS	259	649
< 18.5		2.0	1.5	1.1	1.0			2.4	1.3
18.5–25.0		59.3	30.8	42.0	24.6			45.7	26.5
≥ 25.0 and <30.0		31.4	36.4	42.0	37.6			36.3	36.2
≥ 30.0		7.3	31.3	14.9	36.8			15.6	36.0
Blood pressure (%)	n	149	430	200	550	NS	0.04	275	725
≥ 140/90 mmHg		37.8	23.8	48.7	32.5			44.5	35.1

*NS* non-significant

## Discussion

In the Abena survey carried out in 2011–2012 in French urban zones, food assistance recipients, as expected, showed poor social and living conditions, along with risk of severe food insecurity. Half of the recipients had been receiving food assistance for more than two years and depended entirely on distribution for basic foods such as canned goods and non-perishable products. Accordingly, consumption frequencies of fruit and vegetables, fish and, to a lesser extent, “meat, fish and eggs” and dairy products were low compared to the recommendations, in one-third to one-half of food recipients. Since 2004–2005, changes have been observed in sociodemographic characteristics as well as in food consumption frequencies. Overall, improvement has been observed in consumption frequency of core foods, but is nonetheless limited. The prevalence of obesity and high blood pressure was very high, especially in women.

### Strengths and limitations

To our knowledge, this is the first survey on food assistance in Europe carried out in a large sample at two different time periods, during which public health measures were taken to improve the quality and quantity of foods distributed. In the absence of an experimental procedure, changes observed between the two assessments must be interpreted with caution. The measures taken may have improved the nutritional conditions of food recipients overall, at the population level, but individual characteristics have also changed. Adjusted analyses took into account the latter, but residual confounding may remain. Survey conditions, especially interviews at sites of food distribution, complicated the collection of data such as dietary behavior and nutritional measurements. The short food frequency questionnaire that we used was adapted to that purpose, but comparison with results from more elaborate questionnaires, including amounts eaten for instance [18] or with recommendations [19] is thus limited. In particular, it did not enable deriving nutrient intake for comparisons with previous surveys [20]. Also, sampling was done randomly, but only in six urban zones (four in 2004–2005). Caution must thus be used when attempting to generalize our results to all urban food recipients in France. Other inclusion criteria such as the capacity to speak French (or need for help in interpreting at the time of the interview) may also have interfered with the external validity of our observations. Finally, participation rates have declined between the two surveys, despite using similar protocols. Lack of time was the principal cause for refusal, suggesting that conditions in which participation in the survey was proposed may have changed, due to constraints in the structure organization. Therefore, changes over time should be interpreted cautiously. Besides, participants in 2011–2012 were comparable to non-participants for age (44.1 years ± 13.2 vs. 43.4 ± 13.0) but proportion of participation was higher in men (49.0 %) than in women (41.4 %), which could have led to gender-related biases.

Knowledge of food insecurity, purchasing, dietary intake and nutritional status has improved among participants in the Supplemental Nutrition Assistance Program (SNAP) and the Special SNAP for Women, Infants and Children program (WIC) [21–24]. The latter are not completely identical to programs of food provided by pantries or purchased at charitable grocery stores, since the choice of foods is higher with programs such as SNAP and WIC, although some restrictions exist [25]. Likewise, nutritional information is available to food-insecure households and individuals whether or not they receive assistance [26, 27]. Nonetheless, few surveys have been carried out among randomly sampled individuals who receive food assistance in the form of parcels from pantries or who purchase food at charitable grocery stores [10, 20, 28], and most studies were based on convenient samples in limited settings [8, 29, 30]. Finally, sample sizes were generally very limited (fewer than 500 individuals, vs. >1,000 in our survey), evaluations did not include measurements of weight and height for body weight status estimation and no previous study assessed the core food supply of food assistance recipients. Nevertheless, unlike our survey, some previous studies used comprehensive tools for diet assessment [20].

### Interpretation

Overall, the sociodemographic characteristics and living conditions observed in our survey were consistent with those reported in the publications mentioned above. Most food bank recipients were women, single with or without a child, fairly well educated, unemployed, and who declared high levels of food insecurity. Indeed, using the same USDA 18-item FSSM, moderate to high food insecurity has been previously observed in two-thirds to three-fourths of individuals [10, 21, 28, 30], as in our observations. Time from first use of food assistance has also been described as variable, since it included recipients who had benefitted from such assistance over a long period, while others had only recently done so [10]. Despite variations in the way organizations provide food assistance, sociodemographic characteristics of food recipients are very similar. Specificity concerns the countries of birth, related to the overall background of migration in the various countries.

Frequencies of consumption as assessed in our survey emphasize the risk of insufficient intake of basic foods despite the help provided. While starchy foods were consumed daily by almost all food assistance recipients, dairy foods (16.0 %), fruit and vegetables (18.9 %) and “meat, fish and eggs” (36.9 %) were not consumed daily by more than one person in six. In addition, consumption was lower than recommended in the framework of the French Nutrition and Health Program. This was particularly true for fruits and vegetables, eaten five times a day by less than 10 % of subjects in 2011–2012, and for fish, consumed twice a week or more by only 40 %. These observations are consistent with social disparities observed in the general population [31]. However, compared to observations in 2004–2005 [11], fruits, vegetables and fish were eaten more often in 2011–2012, as was the case for dairy and “meat, fish and eggs”. In France, distribution of such food groups was developed following publication of 2004–2005 survey results, but as yet remains limited, since their acquisition, transportation and storage raise major logistic problems. The observed increase in their consumption may have been partly related to better distribution, since adjustment for socio-demographic changes did not modify results, but other factors may also have interfered.

The obesity problem in food assistance recipients, especially within the context of SNAP and WIC programs, has been widely studied; indeed, very high prevalences have been reported, especially in women [32, 33]. In the Abena survey, the prevalence of female obesity was twice as high as that of the French general population [18]. It was much lower in men, but the prevalence is now closer to what has been reported in the general population [18]. An effect of food assistance, including irregular cycles of food availability, upon the onset of obesity has been hypothesized [34]. Women who restrict their food intake to protect their children’s intake might also be subject to risk of obesity [35]. Our survey also provides original information on high blood pressure, and underlines its high risk, although the age distribution was younger than in the general population. Since 2004–2005, this risk has increased in women but not in men despite a tendency towards an increasing prevalence. Moreover, obesity prevalences have not statistically changed since 2004–2005, despite a trend towards an increase. Thus, in contrast to food consumption, such nutritional markers have not improved since 2004–2005. The role of long-term exposure to an unhealthy lifestyle in the onset of obesity and high blood pressure can be hypothesized: potential improvement related to food consumption changes would be noteworthy only after a much longer period. In addition, overweight and high blood pressure are multifactorial; other factors such as low physical activity, smoking and alcohol intake may not have changed in the meantime.

## Conclusions

The Abena surveys enable follow-up of the nutritional status of adults receiving food assistance in the form of pantry packages or purchases at social grocery stores in France. Despite slight improvements since 2004–2005, figures remained alarming in 2011–2012 regarding low consumption of dairy and fruit and vegetables, while the prevalence of obesity and blood pressure remained high. Food assistance cannot completely compensate for food insecurity and its consequences. Indeed, organizations are encountering major difficulties in providing assistance to all concerned and must solve major logistic and distribution problems. In a context of decreasing social protection for the severely deprived, need for food assistance is increasing, as was documented in the UK [36]. Therefore, given the risk of diseases associated with poor nutrition and, consequently, their health care costs, continued improvement of food assistance quality, quantity and access, is an important public health concern.
